# BCG Vaccination in Early Childhood and Risk of Atopic Disease: A Systematic Review and Meta-Analysis

**DOI:** 10.1155/2021/5434315

**Published:** 2021-11-24

**Authors:** Keyu Zhao, Phoebe Miles, Xinyu Jiang, Qiongyan Zhou, Chao Cao, Wei Lin, Richard Hubbard, Panfeng Fu, Suling Xu

**Affiliations:** ^1^Department of Dermatology, The Affiliated Hospital of Medical School, Ningbo University, 247 Renmin Road, Ningbo 315000, China; ^2^Division of Humanities and Social Sciences, University of Nottingham, 199 Taikang East Road, Ningbo 315000, China; ^3^Department of Respiratory Medicine, Ningbo First Hospital, 59 Liuting Road, Ningbo 315000, China; ^4^Division of Epidemiology and Public Health, University of Nottingham, 199 Taikang East Road, Ningbo 315000, China; ^5^The Center of Medical Research, The Affiliated Hospital of Medical School, Ningbo University, 247 Renmin Road, Ningbo 315000, China

## Abstract

**Background:**

Several large-scale studies suggest that Bacille Calmette–Guerin (BCG) vaccination in early childhood may reduce the risk of atopic diseases, but the findings remain controversial. Here, we aimed to investigate the potential correlation between early childhood BCG vaccination and the risk of developing atopic diseases.

**Methods:**

Eligible studies published on PubMed, EMBASE, and Cochrane CENTRAL were systematically sourced from 1950 to July 2021. Studies with over 100 participants and focusing on the association between BCG vaccine and atopic diseases including eczema, asthma, and rhinitis were included. Preliminary assessment of methods, interventions, outcomes, and study quality was performed by two independent investigators. Odds ratio (OR) with 95% confidence interval (CI) was calculated. Random effects of the meta-analysis were performed to define pooled estimates of the effects.

**Results:**

Twenty studies with a total of 222,928 participants were selected. The quantitative analysis revealed that administering BCG vaccine in early childhood reduced the risk of developing asthma significantly (OR 0.77, 95% CI 0.63 to 0.93), indicating a protective efficacy of 23% against asthma development among vaccinated children. However, early administration of BCG vaccine did not significantly reduce the risk of developing eczema (OR 0.94, 95% CI 0.76 to 1.16) and rhinitis (OR 0.99, 95% CI 0.81 to 1.21). Further analysis revealed that the effect of BCG vaccination on asthma prevalence was significant especially in developed countries (OR 0.73, 95% CI 0.58 to 0.92).

**Conclusion:**

BCG vaccination in early childhood is associated with reduced risk of atopic disease, especially in developed countries.

## 1. Introduction

Allergy is malfunctioned immune response caused by various foreign antigens, which can increase the risk of asthma, atopic dermatitis (eczema), and rhinitis (hay fever) [1]. Atopic diseases are characterized by the incubation period between initial exposure (sensitization) and symptoms (elicitation) that will develop in subsequent exposures and may involve IgE and/or non-IgE-mediated responses. An IgE-mediated allergic reaction (sometimes called immediate-type hypersensitivity (type I)) involves the production of Th2 cytokines, which initiate IgE production by B cells [2]. Increased prevalence in allergic and autoimmune diseases has been appreciated worldwide, especially for the Western countries where the prevalence is as high as 20% [3], and one in three children in these countries [4] are affected by atopic diseases. Atopic diseases also have been positioned as the most common chronic disease, which greatly compromised life quality of patient and impose great social and economic impacts on both individuals and their families. Among patients with atopic disease, pediatric patients are affected greatly as they have high prevalence [5]. A recent register-based study showed that the lifetime prevalence of asthma and allergic rhinitis at age 10 was 15.6% and 20.4%, respectively [6].

Vaccination is used worldwide for preventing infectious diseases [7]. Childhood vaccination plays a crucial role in the early development of the immune system [8]. Atopic disease usually develops in the early life of children, whose immune system is under development [9]. There is evidence revealing the relationship between vaccination and the risk of atopic disease [10], and many publications have investigated this hypothesis [11–15]. Among those relationships, the most investigated one is the relationship between BCG vaccine and atopic disease, but the results remain controversial. National BCG vaccination programs are standard for childhood immunization in most countries apart from Western European counties and North American countries to prevent *tuberculosis* [16]. Th2 immunologic response is inhibited by BCG. Antagonism of atopy by BCG has been observed in both human and animal models. Considering these characteristics, BCG is considered to be a therapeutic model for investigating the consequence of early-life stimulation of TH1 cells [17]. Recently, BCG is shown to have nonspecific beneficial effects on early immune system, reducing infant hospitalization [18]. However there were conflicting results between BCG and childhood atopic disease [19]. To clarify the relationship between BCG and childhood atopic disease, we conducted a meta-analysis of studies. We examined the association between BCG vaccine and atopic disease incidence and explored the implications of available literature data for clinical practice and future examination.

## 2. Methods

### 2.1. Strategy of Literature Search

Our systematic review was performed and reported in accordance with Meta-analysis Of Observational Studies in Epidemiology (MOOSE) [20] and the Preferred Reporting Items for Systematic Reviews and Meta-Analyses (PRISMA) [21] guidelines.

We performed a systematic literature search in PubMed/Medline (1950 to Jul 2021), EMBASE (1980 to Jul 2021), and Cochrane CENTRAL (1950 to Jul 2021) for association between BCG vaccination and atopic disease by using relevant keywords including asthma, eczema, rhinitis, BCG vaccines, and other synonyms. The search strategy suitable for PubMed is provided in Appendix 1. The analysis was restricted to studies published in English. We screened bibliographies of relevant review articles to ensure that all relevant studies were included.

### 2.2. Study Selection

Each study was first selected based on their titles and abstracts (when available) by two independent investigators at the same time. Then, they retrieved full texts and performed further screening when studies were deemed eligible. Studies had to be cohort studies with information of authors, year, geographical area, study design, sample size, exposure (age of BCG vaccination, method of assessment), and outcome (confirmed diagnosis of specific atopic disease). Case reports, preclinical studies, and some studies without confirmed diagnosis of a specific atopic disease will not be used for this study. Disagreements were resolved by discussion and, if necessary, in consultation with a third senior investigator.

### 2.3. Quality Assessment

Quality of all included trials was assessed by two authors independently by using the STROBE checklist for cohort study and JADAD scale for RCT study. The risk of bias in each domain was judged as low, high, or unclear. The overall risk of bias in a study was classified as low if all domains had low risk; as high if one or more domains had high risk; or as unclear otherwise. Based on these standards, we defined the studies into the following three grades: A, high quality and low risk of bias (scored ≥66.6%); B, moderate quality and moderate risk of bias (scored 33.3–66.6%); and C, low quality and high risk of bias (<33.3%). Discrepancy in quality assessment between the reviewers was resolved by discussion with involvement of a third senior investigator if necessary.

### 2.4. Data Extraction

Two authors extracted data independently using a standard data extraction form. The following baseline characteristics were extracted from the included studies: first author, year of publication, study design, and location in which the study was performed, number of included participants, and diagnosis of atopic disease. Studies were excluded if any of the above information is not available.

### 2.5. Statistical Analysis

We used R (version 3.6.3) to perform the data analysis; odds ratios (ORs) and their associated 95% confidence intervals (CI) were used to assess the strength of association between BCG vaccination in early life and the risk of getting atopic disease. Statistical significance was defined at *p* < 0.05 [22]. *I*^2^ statistic were used for investigating heterogeneity, and statistical heterogeneity was defined at *I*^2^ > 50%. We used random-effects modeling to perform the meta-analysis for all pooling. In case heterogeneity exists, analysis to investigate whether the heterogeneity is related to the participant's race will be performed. Different ethnic background in different continents may be considered potentially important to heterogeneity because of living habit diversity. Funnel plots were used to display the publication bias graphically, both specifically and officially with Egger's test.

## 3. Results

**Figure 1:**
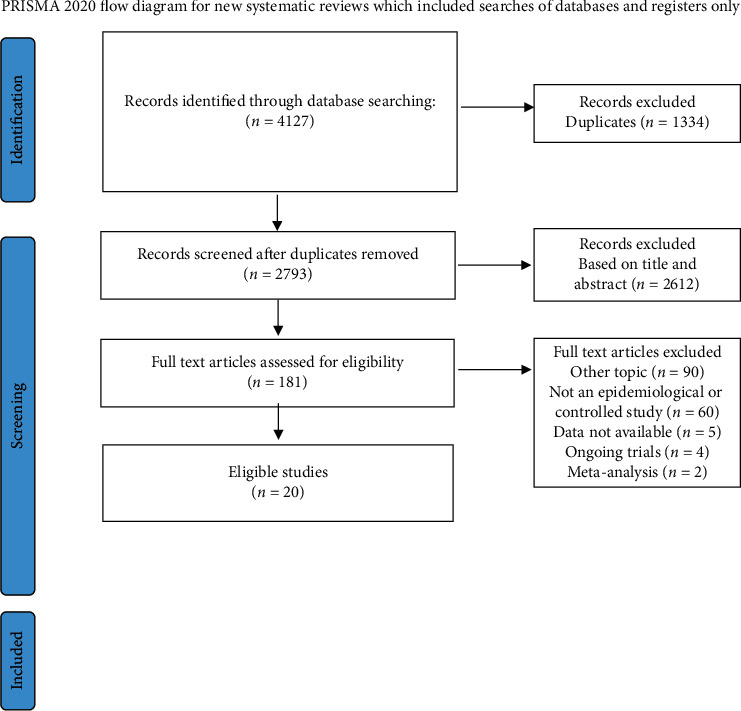
The Preferred Reporting Items for Systematic Review and Meta-Analysis (PRISMA) flow chart for the included studies.

Our search strategy generated 4127 citations from 3 databases. Among them, 3946 articles were removed after exclusion of duplicates and screening of titles and abstracts. Of the remaining 181 studies, 161 studies were excluded after reviewing the full text. In total, 20 articles including 222928 participants met the inclusion criteria and were included in the meta-analysis [11, 23–41].The flow diagram of trial identification and selection is shown in Figure 1. Descriptions and baseline characteristics of included studies are detailed in Table 1. No problems were encountered with participant data deficiency during the data integrity check.

Four studies were conducted in developing countries, and sixteen studies were in developed countries. None of the included studies were at low risk of bias (rated A) as all trials had an element of pragmatism in using different methods. Fifteen studies including 87% participants were deemed to be at moderate risk of bias (rated B). We judged five studies including only 13% participants to have high risk of bias (rated C) in the field of study participation or statistical reporting.

### 3.1. Association of BCG Vaccination in Childhood with Incidence of Atopic Disease

**Figure 2:**
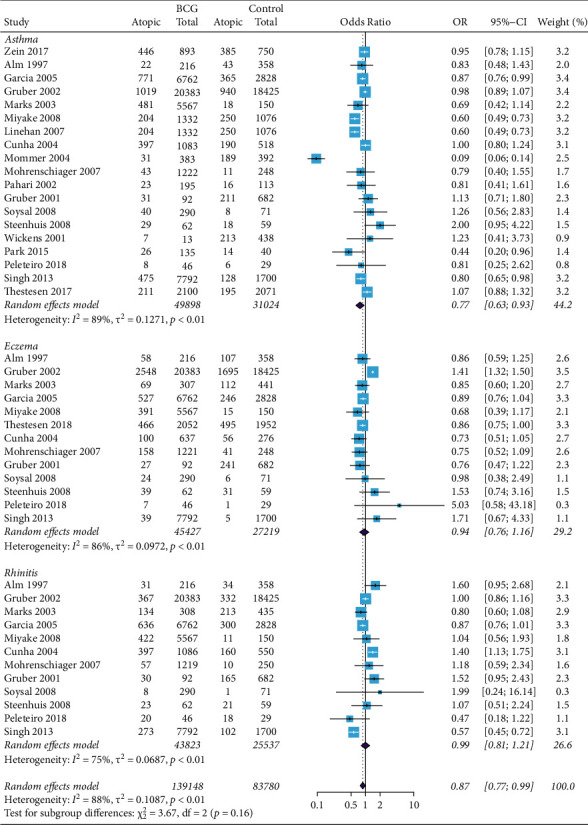
Forest plot of BCG and total atopic diseases. Random effects model forest plot shows ORs and 95% CIs for the association between BCG vaccination and total atopic disease. OR = 0.87, 95% CI 0.77 to 0.99. M-H = Mantel–Haenszel; OR = odds ratio; CI = confidence interval.

In the pooled analysis, we found that participants receiving BCG in childhood associated with a lower risk of atopic disease than that of the non-BCG group (OR = 0.87, 95% CI 0.77 to 0.99; Figure 2). Nineteen studies involving 80922 participants reported the relationship between BCG vaccination and the risk of asthma. Compared with non-BCG group, individuals received BCG in early childhood were associated with a significantly reduced risk of asthma (OR = 0.77, 95% CI 0.63 to 0.93; Figure 2). Thirteen studies including 72646 participants reported the association between eczema and BCG vaccine, and twelve studies including 69360 participants studied rhinitis and BCG vaccine. Compared with the control group, BCG vaccine showed no significant effect on preventing eczema and rhinitis (OR = 0.94 and 0.99, 95% CI 0.76 to 1.16 and 0.81 to 1.21, respectively; Figure 2).

### 3.2. Association between BCG Vaccination and Demographic Factors on the Risk of Atopic Disease

**Figure 3:**
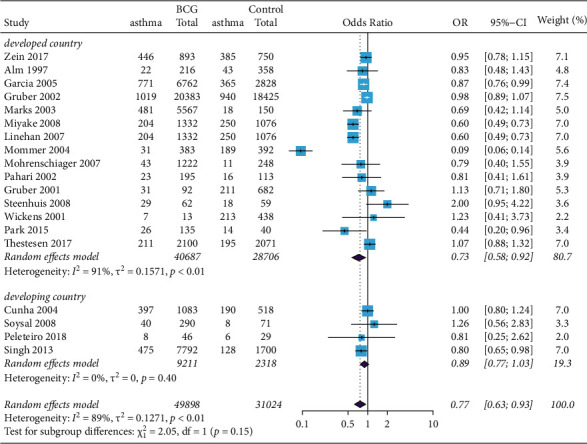
Forest plot of the association between BCG vaccination and asthma. Random effects model forest plot shows ORs and 95% CIs for the association between BCG vaccination and asthma. OR = 0.73, 95% CI 0.58 to 0.92. M–H = Mantel–Haenszel; OR = odds ratio; CI = confidence interval.

We evaluated the association between BCG vaccination and prevalence of asthma in terms of participants' demographics. Participants from developed countries were associated with a significantly lower risk of developing asthma when administered BCG vaccine in early childhood (pooled OR = 0.73, 95% CI 0.58 to 0.92; Figure 3). In contrast, in developing countries, participants who received BCG vaccine in early childhood were not associated with a significantly reduced risk of allergic disease (pooled OR = 0.86, 95% CI 0.69 to 1.07; Figure 3).

**Figure 4:**
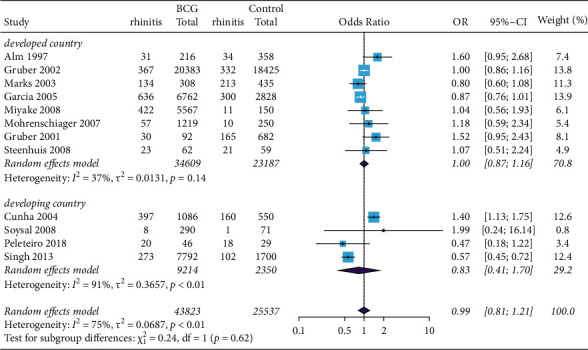
Forest plot of the association between BCG vaccination and rhinitis. Random effects model forest plot shows ORs and 95% CIs of the association between BCG vaccination and rhinitis. OR = 0.99, 95% CI 0.81 to 1.21. M–H = Mantel–Haenszel; OR = odds ratio; CI = confidence interval.

**Figure 5:**
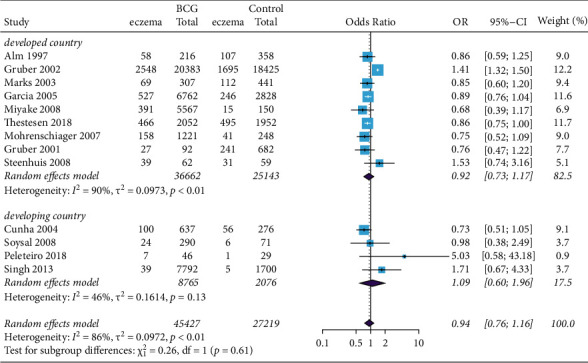
Forest plot of the association between BCG vaccination and eczema. Random effects model forest plot shows ORs and 95% CIs for the association between BCG vaccination and eczema. OR = 1.09, 95% CI 0.60 to 1.96. M–H = Mantel–Haenszel; OR = odds ratio; CI = confidence interval.

BCG vaccine was not associated with the risk of eczema in the subgroup analysis of different continents (Figure 4). Similar results were obtained in participants with rhinitis (Figure 5). These results of our study did not support an association of BCG vaccination with reduction in the risk of eczema or rhinitis in both developed and developing countries.

### 3.3. Publication Bias

**Figure 6:**
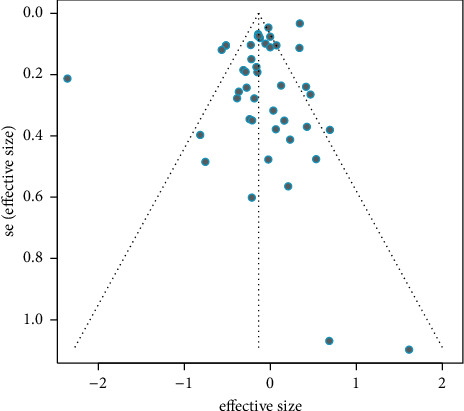
Funnel plot for the association between BCG vaccination and total atopic diseases. See Table 1 for reference citations. SE = standard error. *p*=0.074.

The total publication bias was outlined in the funnel plot (Figure 6). Based on the funnel plots and Egger's test [42], it suggested that publication bias did not impact our estimates (*p*=0.074).

## 4. Discussion

In this systematic review and meta-analysis, we revealed that BCG vaccine might be effective in preventing atopic disease, especially for asthma among developed countries. However, compared with asthma, the risk of developing atopic disease was not reduced for both eczema and rhinitis. In developing countries, there was no association between BCG vaccine and atopic diseases. As far as we know, this systematic review and meta-analysis is the latest study investigating the association between BCG vaccination and atopic diseases.

In this study, we found that receiving a BCG vaccine in early childhood especially reduced the risk of asthma. According to other studies on BCG vaccination and asthma, BCG vaccine, which was proved to prevent the inflammation caused by mycobacteria, has been tested to inhibit allergen-induced airway inflammation in a murine model of atopic asthma [43]. The immune balance was adjusted by BCG vaccine during Th1-like activity and decreasing the IL-4 and IL-10 production. It was not clear how IFN-*γ* influences Th2 responses, but it may involve macrophage activation and inhibition of Th2 lymphocytes development or change antigen presentation directly [44]. The mechanism of BCG vaccine on asthma was not as simple as changes in the Th1/Th2 balance [45], and we speculated that early childhood BCG vaccination accelerates the conversion from a Th2 to a Th1 type, therefore restraining the expression of atopy.

In developed countries, the association between BCG vaccination and reduction of the risk of asthma is more significant than the other developing countries of the world. This could be attributable to the fact that, in the regions other than developed countries, helminth infections and *tuberculosis* are endemic; therefore, the effects of BCG vaccine on reducing prevalence of allergic diseases were compromised [46]. The protection level of BCG vaccination is correlated to the gradient of exposure to environmental mycobacteria, a gradient from lower protection in countries close to the equator towards higher protection with increasing distance from the equator [47]. It is also reported that early-life events or diseases like perinatal circumstances or early allergen exposure would increase the prevalence of atopic diseases [48]. However, due to the limited number of studies included, heterogeneity might not be shown in the subgroup analysis of subjects at high risk or of developing countries. Based on this meta-analysis, the positive protective role of BCG vaccine in atopic disease requires further investigation, especially more cohort studies on children from high-risk areas.

As for rhinitis and eczema, there is no significant association, even though they may share similar genetic characteristics with asthma [49]. This is likely because some methodologic limitations may limit our interpretation of the findings. We expected that analyzing heterogeneity through technical means to analyze the original data might be helpful to rule out the reasons of the heterogeneity generated by specific studies. Due to the lack of information on the severity of atopic diseases, especially for eczema in the included studies, the applicability of the findings of children with varying degrees of severity is therefore compromised. This may have influenced the resulting protective effects of BCG vaccination in eczema and rhinitis.

Our meta-analysis has several strengths. Compared with a similar meta-analysis [19], six new articles were included in this paper. Each study sample size in this meta-analysis was assessed by two independent authors, so the result can be more accurate than others with low quality [50]. Furthermore, we followed the recommendations of the Cochrane Collaboration and PRISMA statement, including a priori protocol. Comprehensive assessment of the study quality was achieved by using STROBE checklist for the cohort studies and JADAD scale for the RCT study.

As with all systematic reviews, we may have failed to identify some studies, especially those with negative results, several studies are missing in this meta-analysis, including RCTs concerning neonatal BCG vaccination and wheeze and other possibly atopic diseases. Therefore, this may have influenced our findings. Finally, the length of time that the early effective protection from BCG vaccination lasts remains unanswered. The age of participants in this review may partially explain the result of the protective effect of BCG vaccination. In Linehan's study, it was shown that any benefits of BCG vaccine are likely to be transient [51]. Therefore, a large proportion of the protection from BCG vaccination may not be attributed to a reduction in the risk of atopy. Nonetheless, it can be confirmed that BCG vaccination in early childhood does reduce the risk of developing asthma in early life.

## 5. Conclusion

Our results provide evidence that BCG protects against the risk of atopic diseases with the most protective effects on asthma occurrence from multiple epidemiologically different settings. Our results also suggest that that BCG vaccination in early childhood is associated with reduced risk of atopic disease, especially in developed countries.

## Figures and Tables

**Table 1:** Characteristics of included studies.

Reference (year of publication)	Design	Population studied	Country	Sample size (case/control)	Outcomes and method	Quality assessment
Zein 2017	Retrospective study	Vaccination: 32,900 (42.9%) individuals received the vaccine within their first year of life, 2,712 (3.5%) were vaccinated later. Follow up: children born at 32 weeks of gestation until 20n years Quebec, Canada, 1974.	Developed country	893/750	Asthma at least 2 asthma-related medical services or at least 1 asthma- related hospitalization (international classification of diseases, ninth revision, code 493)	B

Alm 1997	Retrospective study	Vaccination: Before the age of 6 months, follow up: children at 3–7 years Stockholm, Sweden 1991	Developed country	216/358	Eczema, asthma, rhinitis atopic disease in history and/or at the clinical examination, as well as skin prick test and IgE in a blood sample	B

Garcia 2005	Retrospective study	Vaccination: When the children were born follow up: children aged 6 and 7 years old health centers in Spain	Developed country	6762/2828	Eczema, asthma, rhinitis the Spanish version of the ISAAC phase III questionnaire	B

Gruber 2002	Retrospective study	Vaccination: When the children were born follow up: children who were starting school (mean age 6 years) Germany, 1994	Developed country	20383/18425	Eczema, asthma, rhinitis a mandatory health survey	B

Marks 2003	Retrospective study	Vaccination: In the first 8 weeks of life follow up: yellow race children aged 7 to 14 years old Sydney, Australia	Developed country	309/442	Eczema, asthma, rhinitis subjects underwent allergen SPTs, spirometry, a methacholine inhalation challenge test, a venous blood collection for IgE assay and lymphocyte studies, and a TST	B

Miyake 2008	Retrospective study	Vaccination: In infancy according to school records follow up: 8–11 years of age Japan	Developed country	5567/150	Eczema, asthma, rhinitis a self-administered questionnaires included questions on symptoms of wheeze, asthma, atopic eczema	B

Linehan 2007	Retrospective study	Vaccination: Before the age of 12 weeks followed up: 6–11 years of age health care center, England	Developed country	1332/1076	Wheeze a parent-completed questionnaire, based on the international study of asthma and allergies in childhood asthma questionnaire 12 with some additional questions	B

da cunha 2004	Cross-sectional	Vaccination: When the children were born followed up: children of 12–14 years Salvador, Brazil	Developing country	1089/523	Eczema, asthma, rhinitis based on self-reporting of current allergy and whether this was accompanied by sneezing and/or skin manifestations	C
Mommers 2004	Nested case-control study	Vaccination: When the children were born followed up: children at 7–8 years of age Netherlands and Germany	Developed country	Case:75	Asthma ISAAC questionnaire and questions on indoor environment, specific IgE was measured in the blood	C

Mohrenschlager 2007	Cross-sectional study	Vaccination: Unknow followed up: at 5–7 years (preschool) augsburg Germany	Developed country	1219/247	Eczema, asthma, rhinitis skin prick test reaction more than 2 mm	C

Pahari 2002	Cross-sectional study	Vaccination: Unknow follow up: children aged 11–18 years from secondary school England	Developed country	Case:308	Asthma clinical symptom	C

Thestesen 2018	RCT	Vaccination: When the children were born follow up: from birth to 13 months of age Denmark	Developed country	2052/1952	Eczema the severity of AD was graded using SCORAD	B

Gruber 2001	Prospective study	Vaccination: Within their first weeks of life follow up: from birth to 7 years old Freiburg, Germany	Developed country	92/682	Eczema, asthma, rhinitis parents filled in a questionnaire and gave a structured interview about their children *s* diseases and atopic symptoms	B

Soysal 2008	Retrospective study	Vaccination: Unknow follow up: children with recent household contact to adult given a diagnosis of sputum smear– positive pulmonary TB at one of 7 government- funded TB clinics istanbul	Developing country	290/71	Eczema, asthma, rhinitis all children underwent TST and chest radiography, and gave 10-ml of venous blood sample for the RD1 ELISpot assay and total serum IgE level	B

Steenhuis 2008	RCT	Vaccination: When the children were born Follow up: high-risk newborns in the pediatric Utrecht, Netherlands	Developed country	62/59	Eczema, asthma rhinitis an adapted version of the british medical research council questionnaire and the Dutch version of the european community respiratory health survey were used	C

Wickens 2001	Case-control study	Vaccination: When the children were born follow up: children aged 6–7 and 13–14 from a random selection of schools Wellington, New Zealand	Developed country	220/231	Asthma the case-control study was based on the Wellington, New Zealand, arm of the ISAAC	C

Peleteiro 2018	RCT	Vaccination: All individuals had been vaccinated with BCG in early childhoodFollow up: healthy undergraduate studentsSalvador, Bahia, Brazil	Developing country	46/29	Eczema, asthma rhinitis sociodemographic and clinical characteristics were obtained using a standard questionnaire and the ISAAC questionnaire. Total serum IgE levels were measured	B
Park 2015	Retrospective study	Vaccination: unknowFollow up: adults who underwent skin pricktesting, Korea	Developed country	135/40	Asthma the results from spirometry and bronchial provocation testing conducted within one month from the date of SPT	C

Singh 2013	Cross-sectional study	Vaccination: unknowFollow up: aged 7–14 years Chandigarh, North India.	Developing country	7792/1700	Eczema, asthma rhinitis symptoms were assessed according to the ISAAC phase II questionnaire that asked for infor- mation on demography, symptoms of wheeze, asthma, rhinitis, and eczema, together with various possible risk factors	B

Thestesen 2017	RCT	Vaccination: When the children were born follow up: newborns (3–13 months) Denmark	Developed country	2100/2071	Asthma at 3 and 13 months, the children were invited for a clinical examination at the study site, where study staff evaluated the child's breathing and made an auscultation	B

## Data Availability

All data generated or analyzed during this study are included in this published article and its supplementary information files.

## Abbreviations

BCG: Bacille Calmette–Guerin
CI: Confidence intervals
OR: Odds ratios
PRISMA: Preferred Reporting Items for Systematic Reviews and Meta-Analyses
CENTRAL: Cochrane Central Register of Controlled Trials
MOOSE: Meta-Analysis of Observational Studies in Epidemiology
IL: Interleukin
TH: T-helper cell.
