# Supplementary material for: BCG Vaccination in Early Childhood and Risk of Atopic Disease: A Systematic Review and Meta-Analysis
Source: Can Respir J. 2021 Nov 24;2021:5434315. doi: 10.1155/2021/5434315 (PMC8635936; doi:10.1155/2021/5434315)
Supplement: Supplementary Materials — The search strategy suitable for PubMed is provided in Appendix 1. The PRISMA checklist includes the Systematic Reviews and Meta-Analyses (PRISMA) guidelines. [file 5434315.f1.zip › 5434315.f1/appendix1 (1).docx]

1. BCG Vaccine/
2. BCG vaccin∗.mp. [mp=title, original title, abstract, name of substance word, subject heading word]
3. Bacillus Calmette-Guerin vaccin∗.mp. [mp=title, original title, abstract, name of substance word, subject heading word]
4. BCG immuni?ation.mp. [mp=title, original title, abstract, name of substance word, subject heading word]
5. tuberculin response.mp. [mp=title, original title, abstract, name of substance word, subject heading word]
6. 1 or 2 or 3 or 4 or 5
7. Asthma/
8. asthma.mp. [mp=title, original title, abstract, name of substance word, subject heading word]
9. night cough∗.mp. [mp=title, original title, abstract, name of substance word, subject heading word]
10. Respiratory Hypersensitivity/
11. bronchial disorder.mp. [mp=title, original title, abstract, name of substance word, subject heading word]
12. hyper-responsiveness wheez∗.mp. [mp=title, original title, abstract, name of substance word, subject heading word]
13. wheez∗.mp. [mp=title, original title, abstract, name of substance word, subject heading word]
14. allergic alveolitis.mp. [mp=title, original title, abstract, name of substance word, subject heading word]
15. Respiratory Sounds/
16. Or/7-15
17. Dermatitis, Atopic/
18. Eczema/
19. Neurodermatitis/
20. eczema.mp. [mp=title, original title, abstract, name of substance word, subject heading word]
21. dermatiti∗.mp. [mp=title, original title, abstract, name of substance word, subject heading word]
22. eczematous edg3 dermatiti∗.mp. [mp=title, original title, abstract, name of substance word, subject heading word]
23. besnier∗ prurigo.mp. [mp=title, original title, abstract, name of substance word, subject heading word]
24. prurigo.mp. [mp=title, original title, abstract, name of substance word, subject heading word]
25. pruritus.mp. [mp=title, original title, abstract, name of substance word, subject heading word]
26. itching.mp. [mp=title, original title, abstract, name of substance word, subject heading word]
27. neurodermatitis.mp. [mp=title, original title, abstract, name of substance word, subject heading word]
28. Urticaria/
29. urticaria.mp. [mp=title, original title, abstract, name of substance word, subject heading word]
30. Or/17-29
31. Rhinitis/
32. Rhinitis, Allergic, Seasonal/
33. Rhinitis, Allergic, Perennial/
34. rhiniti∗.mp. [mp=title, original title, abstract, name of substance word, subject heading word]
35. hayfever.mp. [mp=title, original title, abstract, name of substance word, subject heading word]
36. hay edg3 fever.mp. [mp=title, original title, abstract, name of substance word, subject heading word]
37. poll?nosis.mp. [mp=title, original title, abstract, name of substance word, subject heading word]
38. pollen edg3 allerg∗.mp. [mp=title, original title, abstract, name of substance word, subject heading word]
39. Nasal Obstruction/
40. Conjunctivitis/
41. Conjunctivitis, Allergic/
42. conjunctivit∗.mp. [mp=title, original title, abstract, name of substance word, subject heading word]
43. rhino-conjunctivit∗.mp. [mp=title, original title, abstract, name of substance word, subject heading word]
44. rhinoconjunctivit∗.mp. [mp=title, original title, abstract, name of substance word, subject heading word]
45. Or/31-44
46. Hypersensitivity/
47. allerg∗.mp. [mp=title, original title, abstract, name of substance word, subject heading word]
48. react∗ edg3 allerg∗.mp. [mp=title, original title, abstract, name of substance word, subject heading word]
49. atop∗.mp. [mp=title, original title, abstract, name of substance word, subject heading word]
50. Or/46-49
51. Anaphylaxis/
52. anaphylaxis react∗.mp. [mp=title, original title, abstract, name of substance word, subject heading word]
53. anaphylactic react∗.mp. [mp=title, original title, abstract, name of substance word, subject heading word]
54. anaphylactic shock∗.mp. [mp=title, original title, abstract, name of substance word, subject heading word]
55. anaphylactoid syndrome∗.mp. [mp=title, original title, abstract, name of substance word, subject heading word]
56. anaphylactoid shock∗.mp. [mp=title, original title, abstract, name of substance word, subject heading word]
57. acute systemic allergic react∗.mp. [mp=title, original title, abstract, name of substance word, subject heading word]
58. idiopathic anaphylaxis.mp. [mp=title, original title, abstract, name of substance word, subject heading word]
59. Or/51-58
60. sensiti?ation.mp. [mp=title, original title, abstract, name of substance word, subject heading word]
61. skin prick test.mp. [mp=title, original title, abstract, name of substance word, subject heading word]
62. RAST.mp. [mp=title, original title, abstract, name of substance word, subject heading word]
63. Radioimmunosorbent Test/
64. Radioallergosorbent Test/
65. Immunoglobulin E/
66. specific IgE.mp. [mp=title, original title, abstract, name of substance word, subject heading word]
67. Or/60-66
68. Epidemiologic Studies/
69. Cohort Studies/
70. Case-Control Studies/
71. cohort stud∗.mp.
72. case-control stud∗.mp.
73. etiology.mp.
74. trial.mp.
75. Clinical Trial/
76. clinical trial.mp.
77. Controlled Clinical Trial/
78. controlled clinical trial.mp.
79. Randomized Controlled Trial/
80. randomi?ed controlled trial.mp.
81. Or/68-80
82. 16or30or45or59or67
83. 6and81and82
